# Self-organization of gold nanoparticles on silanated surfaces

**DOI:** 10.3762/bjnano.6.242

**Published:** 2015-12-10

**Authors:** Htet H Kyaw, Salim H Al-Harthi, Azzouz Sellai, Joydeep Dutta

**Affiliations:** 1 Physics Department, College of Science, Sultan Qaboos University, P.O. Box 36, Al-Khoudh, Muscat 123, Sultanate of Oman; 2 Chair in Nanotechnology, Water Research Center, Sultan Qaboos University, PO Box 17, Al-Khoudh, Muscat 123, Sultanate of Oman; 3 Functional Materials Division, School of Information and Communication Technology, KTH Royal Institute of Technology, Isafjordsgatan 22, SE-164 40 Kista Stockholm, Sweden

**Keywords:** 3-aminopropyletriethoxysilane, electrostatic interaction, functionalization, gold nanoparticles, self-assembled monolayer, self-organization

## Abstract

The self-organization of monolayer gold nanoparticles (AuNPs) on 3-aminopropyltriethoxysilane (APTES)-functionalized glass substrate is reported. The orientation of APTES molecules on glass substrates plays an important role in the interaction between AuNPs and APTES molecules on the glass substrates. Different orientations of APTES affect the self-organization of AuNps on APTES-functionalized glass substrates. The as grown monolayers and films annealed in ultrahigh vacuum and air (600 °C) were studied by water contact angle measurements, atomic force microscopy, X-ray photoelectron spectroscopy, UV–visible spectroscopy and ultraviolet photoelectron spectroscopy. Results of this study are fundamentally important and also can be applied for designing and modelling of surface plasmon resonance based sensor applications.

## Introduction

Molecular self-assembly or self-organization is a technique which is widely used for the spontaneous arrangement of nanomaterials. Coupling agents such as thiol-terminated, amine-terminated, alkyl-terminated and phenyl-terminated silanes on metal nanoparticles (so called adsorbates) attach on various substrates through their terminal groups [1–4]. Among them, aminopropyltriethoxysilane (APTES; NH_2_(CH_2_)_3_Si(OCH_2_CH_3_)_3_) is probably the best-known coupling agent used for surface functionalization. APTES has three hydrolysable ethoxy groups that attach to the metal nanoparticle surface (known as silanization process) and the amine (NH_2_) from the aminopropyl groups (pointing away from the surface) for further functionalization [5]. APTES has been extensively studied, and it has been reported that the orientation and attachment of APTES on surfaces depend on temperature, humidity, concentration of APTES and deposition time [6]. To achieve NH_2_ groups as the interface between APTES molecule and nanoparticles is a major issue in silanization processes. During the self-assembly process, terminal amine (NH_2_) can be folded to form hydrogen bonds with free silanol groups, which leads to multilayer deposition of APTES molecules [7]. Thus, NH_2_-terminated APTES deposition on any substrate is extremely important for further surface modification.

Gold nanoparticles (AuNPs) have unique physical, chemical and electrical properties that differ from the bulk due to the quantum confinement effects in small structures [8]. AuNPs have been studied intensively for a wide range of applications such as catalysis [9], biosensing [10], colorimetric sensing [11], optical sensing (surface plasmon resonance (SPR) and surface-enhanced Raman scattering (SERS)) [12–13], photonics [13–14], photovoltaic devices [15] and photothermal therapy [16]. AuNPs exhibit well-defined optical properties such as surface plasmon resonance, which depends on the size and shape of nanoparticles, interparticle distance and the effective refractive index of the surrounding medium [17]. Different techniques have been used to assemble AuNPs on various surfaces in which the stability of AuNPs, interface layer and the support surface are important for the self-organization. Han et al. have demonstrated the growth of a single layer AuNPs film deposited on indium tin oxide (ITO) substrate without using an adhesive layer by utilizing diblock copolymer micelle and spin casting technique [18], while Acik et al. reported in situ deposition of AuNPs on ITO and glass substrate by using spray pyrolysis (temperature range of 260–400 °C) [19]. Wu et al. proposed AuNPs deposition by centrifugation where the thickness depended on centrifugation time [20]. A considerable amount of work has focused on the immobilization of AuNPs on amine-terminated self-assembled monolayer on different substrates [21–23].

In this study, colloidal self-organization of AuNPs was achieved through electrostatic interactions between AuNPs and the functionalized surface. To the best of our knowledge, this is the first report and intensive analysis of self-organisation of AuNPs depending on different orientations of self-assembled APTES molecules on glass substrates. This was studied by observing coverage of AuNPs on glass substrate, difference in surface wetting and changes in electronic and optical properties. Furthermore, orientation and shape of AuNPs deposited on glass substrates were changed upon annealing, which is also reported.

## Results and Discussion

### X-ray photoelectron spectroscopy (XPS) analysis of APTES-modified glass substrates

**Figure 1:**
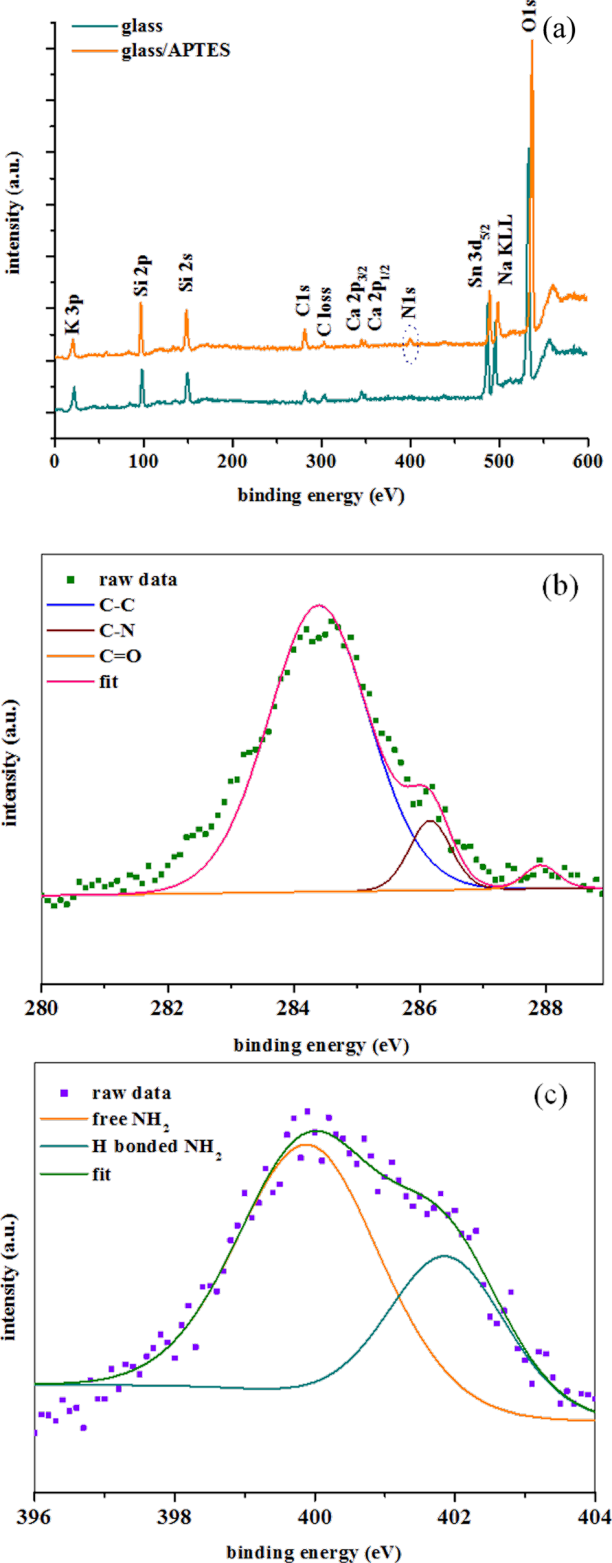
(a) XPS survey spectra of 1% (v/v) APTES-functionalized glass substrate (24 h); (b) high resolution XPS spectra of C 1s and (c) N 1s after functionalization with APTES molecules.

XPS spectra recorded before and after functionalization of glass substrates for 24 h with 1% APTES are shown in Figure 1. The survey spectra (Figure 1a) show oxygen, carbon, silicon, tin, potassium, calcium and sodium KLL peaks of non-functionalized glass substrates as well as APTES-modified glass substrates (The core peak of Na 1s was observed at 1071 eV and is not shown in Figure 1a). A nitrogen peak was, however, not observed on the bare glass substrate. However, a binding energy of 400 eV, which arises from a nitrogen component, could be found on APTES-modified glass substrate. Additionally an increase of the C 1s peak was observed after APTES deposition on glass substrate. Figure 1b,c show the high resolution scan of C 1s and N 1s peaks of APTES-modified glass. Three contributions, namely C 1s peaks that can be assigned to C–C at 284.4 eV, C–N at 286.2 eV and C=O at 288 eV, respectively, are observed. All these signals clearly point to the presence of APTES (C_9_H_23_NO_3_Si) on the glass substrates as it is confirmed with the presence of N 1s peak (Figure 1c). A peak arising at 399.9 eV can be attributed to amine groups while the peak at 401.9 eV can be assigned to the protonated amines (Figure 1c) [24].

### Self-assembled orientation of APTES molecules on glass substrate surface

**Figure 2:**
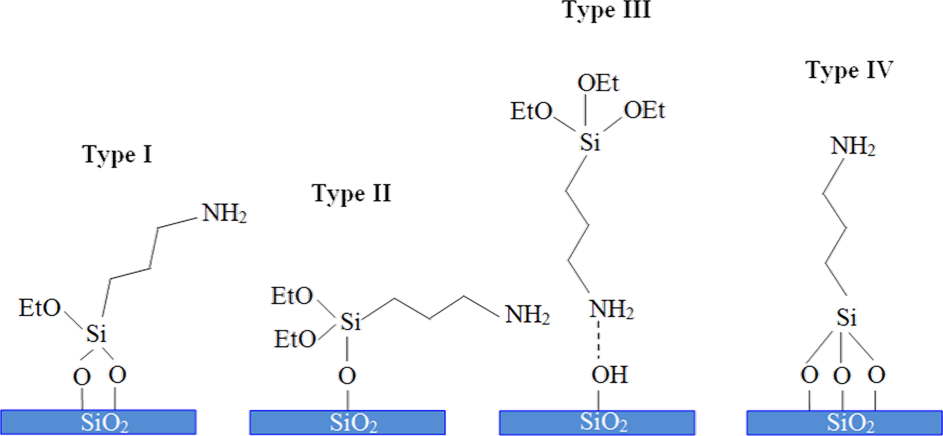
Schematic representation of the possible orientations of APTES molecules on -OH terminated glass substrates. The substrates were functionalized with 1% (v/v) APTES for 24 h.

Four possible types of interfaces between an APTES molecule and the glass surface can be obtained during self-assembly as shown in Figure 2. One or two of the ethoxy groups from the APTES molecules bonded with hydroxylated glass (type I and type II) may create cross-linking of APTES molecules, which in turn would lead to lower availability of free amine terminal groups for a potential attachment of AuNPs. Another possible scenario (type III) is that hydrogen bonds may form between some of the amine functional groups and –OH from the hydroxylated glass substrate leading the –NH_2_ terminal group towards the substrate [24–25]. If APTES molecules are perfectly adsorbed on the –OH terminated glass substrate, a full coverage of AuNPs on the silanized glass substrate can be achieved. In this scenario, each Si bond from the APTES molecules would be covalently bonded to the oxygen from the SiO_2_ surface as a tripod structure (shown as type IV) with higher available amino groups oriented away from the substrate thus being available for binding gold nanoparticles [24]. These different types of orientation of APTES molecules could play an important role to change the surface wetting properties of the glass substrates leading to strong or weak bonding between APTES and metal nanoparticles like Au or Ag [25–26].

### Wetting behavior of APTES-functionalized glass substrates

**Figure 3:**
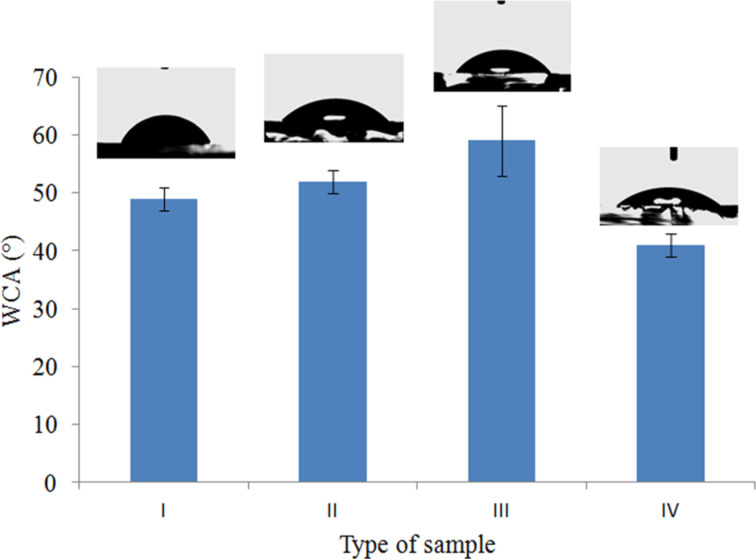
Water contact angles showing wetting behavior of APTES-functionalized glass substrates.

Water contact angle (WCA) measurements were performed to study the surface wetting behavior of APTES-functionalized glass substrates. The corresponding WCAs are shown in Figure 3. Four different types of surface wetting properties were observed on the same APTES-functionalized substrate surfaces: hydrophilic surface with WCA of ca. 41° (Figure 3, type IV) was obtained due to the formation of a silane layer on the glass substrate terminated with free hydrophilic amine groups (see Figure 2, type IV). In this case, hydrophilic amine groups dominated on the glass surface. The WCA of ca. 49° and 52° for type I and II samples were due to the partial exposure of hydrophobic hydro-carbons and hydrophilic –NH_2_ functional groups (See Figure 2). However, in the case of type III samples, higher WCA of ca. 59° was observed which could be due to hydrogen bonding of –NH_2_ with –OH terminated silica groups whereby the glass surface was mainly exposed with hydro-carbon group (EtO) from the silanes (Figure 2, type III).

### Surface morphology analysis of Au nanoparticles deposited on APTES-functionalized glass substrates

**Figure 4:**
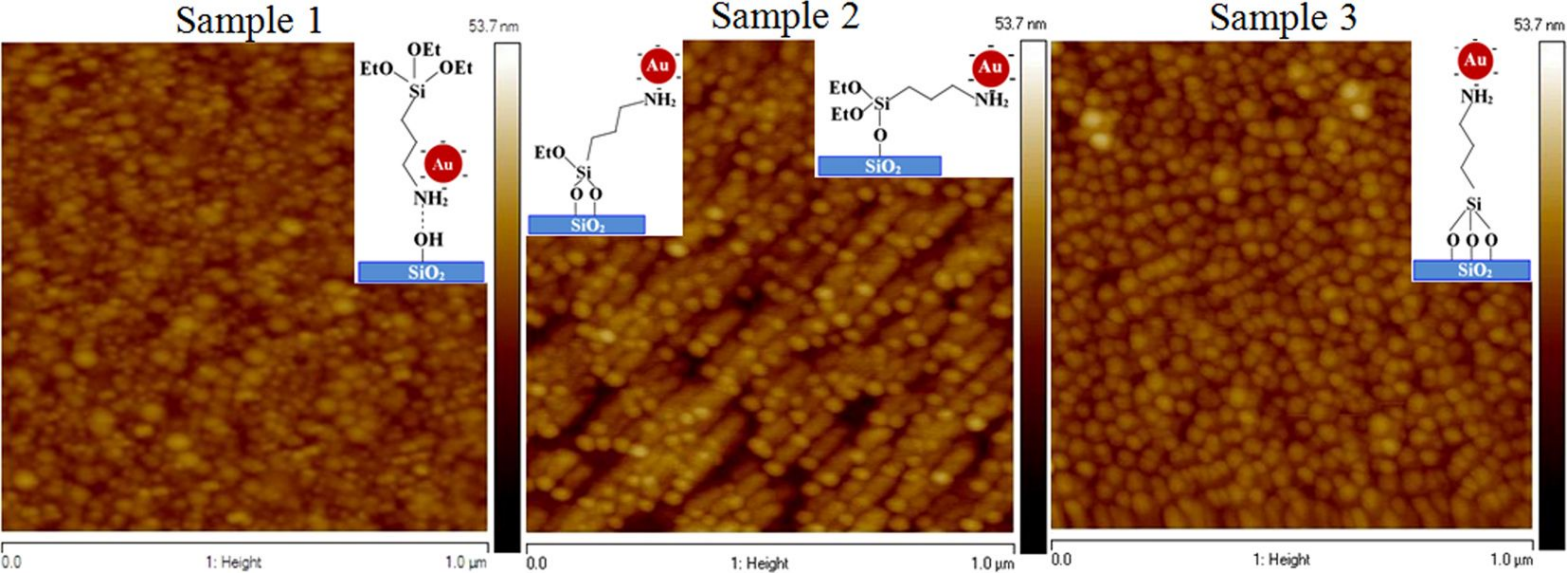
Different surface morphologies of AuNPs attachment on APTES-functionalized glass substrates.

The surface morphology of AuNPs deposited on self-assembled APTES-functionalized glass substrates (functionalization time of 24 h) are shown in Figure 4. The surface morphology of AuNPs on APTES-functionalized glass substrates was followed the randomized orientation of APTES (type I, II III and IV) as described before. Three different surface morphologies of AuNPs deposition are being assigned as sample 1, 2 and 3 (Figure 4). Partial coverage of AuNPs on sample 1 and 2 were observed, but homogenous coverage of AuNps can be seen in sample 3. It is known that AuNPs can be bonded to APTES through the binding of the amine group (–NH_2_) [27]. The possible orientation of APTES molecules shown in Figure 2 can be correlated with AFM images. The lower coverage of AuNPs as observed in sample 1 is probably due to a lower number of free –NH_2_ terminal groups as well as the dominant orientation of APTES molecules attached on glass substrate as mentioned earlier while describing the type III substrate in Figure 2. Slight improvement in the coverage of AuNPs is observed in sample 2 due to the orientation of APTES molecules favoring the attachment of metal nanoparticles as described in Figure 2 (type I, II). However, NH_2_-terminated silanized glass (Figure 2, type IV) surfaces could lead to large amount of AuNPs attached to the amine groups through electrostatic interaction as shown in sample 3.

### Ultraviolet photoelectron spectroscopy (UPS) of AuNPs deposited on silanized glass substrates

**Figure 5:**
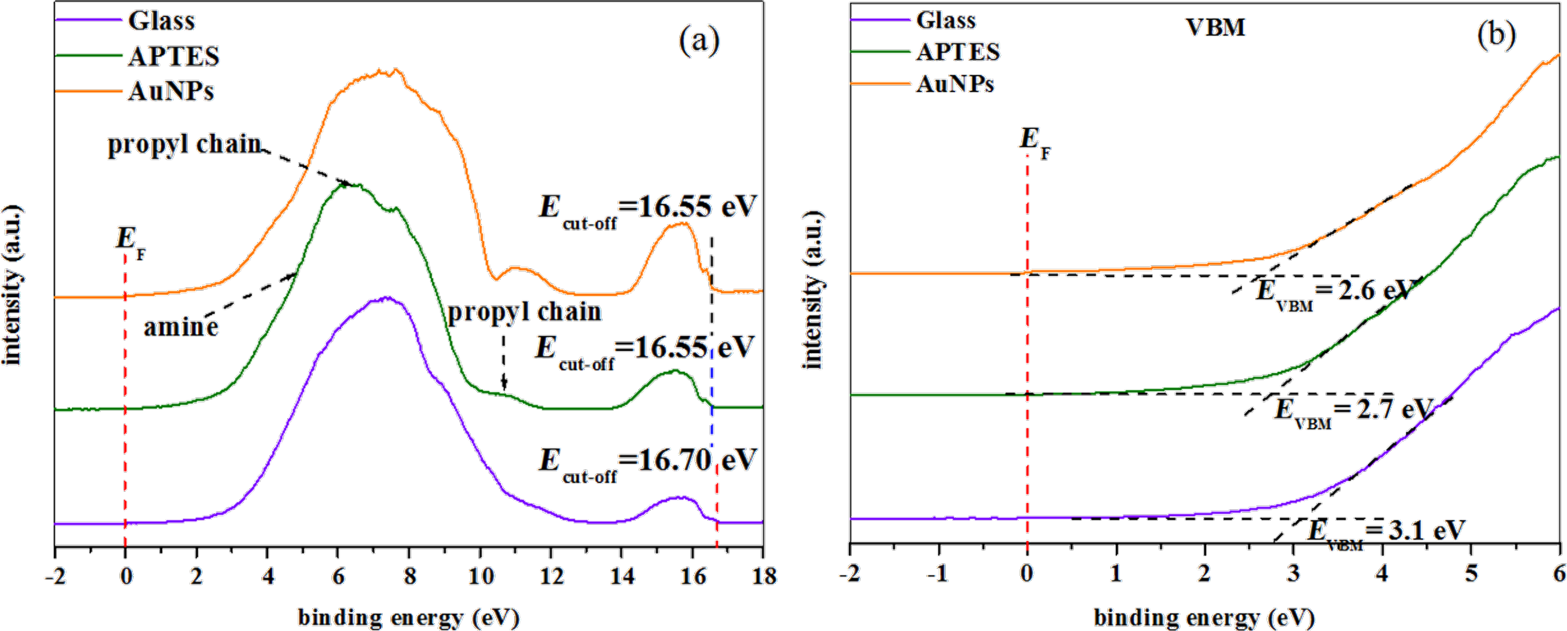
(a) UPS spectra of bare glass, APTES deposited on glass substrate and AuNPs deposited on APTES-functionalized glass substrate (b) the valance band maximum of samples (bare glass, APTES deposited glass and Au NPs deposited glass surface) obtained by linear extrapolations.

The UPS valence band spectra of the bare glass substrate, 1% (v/v) APTES deposited on glass substrate and AuNPs deposited on APTES-functionalized glass substrate are shown in Figure 5a. The maximum peak (λ_max_) intensity on glass surface is observed at 7.4 eV. Upon APTES deposition, λ_max_ is shifted to lower binding energies of ca. 6.3 eV, which can be attributed to the propyl chains (propyl 1) and a peak observed at 10.6 eV can be assigned to the propyl chain (propyl 2) of the APTES molecule [24]. Upon the deposition of AuNPs on APTES-functionalized glass substrate, the λ_max_ intensity shifted back to its original binding energy of pure glass surface, 7.4 eV. This suggests that electrons are donated from AuNPs to APTES molecules through an electrostatic interaction during the attachment to the glass surface. Due to the electron transfer from AuNPs to APTES molecules, slight binding energy shift with higher intensity of propyl chain (from 10.6 eV to 11 eV) can be observed upon the deposition of AuNPs on APTES-functionalized glass substrate (Figure 5a) . However, the amine peak of APTES at 4.8 eV [24] could not be observed due to the interference from the glass substrate. The features observed at ca. 16 eV are due to the background of secondary electron emissions which arise from the substrates near-surface region. An enhancement in the spectral intensity is observed at the secondary electron emission after APTES was deposited on the glass substrate and AuNPs were deposited on the APTES-functionalized glass substrate. The work function (Φ) can be calculated from the difference in the photon energy of He(I) (21.2 eV) and the energy difference Δ*E* between the secondary cut-off energy (*E*_cut-off_) and the Fermi edge (*E*_F_) (as shown in Figure 5a) [28] as


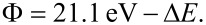


The work function is obtained as 4.5 eV for glass and 4.65 eV for the APTES-functionalized glass substrate as well as the substrate with AuNPs deposited on APTES-functionalized glass. The valance band maximum (VBM) of bare glass, APTES deposited on glass and AuNPs deposited on the glass surfaces were obtained from the linear extrapolations of the foremost edges of the UPS data graphs (Figure 5b). The valence band maximum (*E*_VBM_) is found to decrease from 3.1 to 2.7 eV after APTES was deposited on glass and to 2.6 eV after AuNPs were deposited on APTES-functionalized glass substrate.

**Figure 6:**
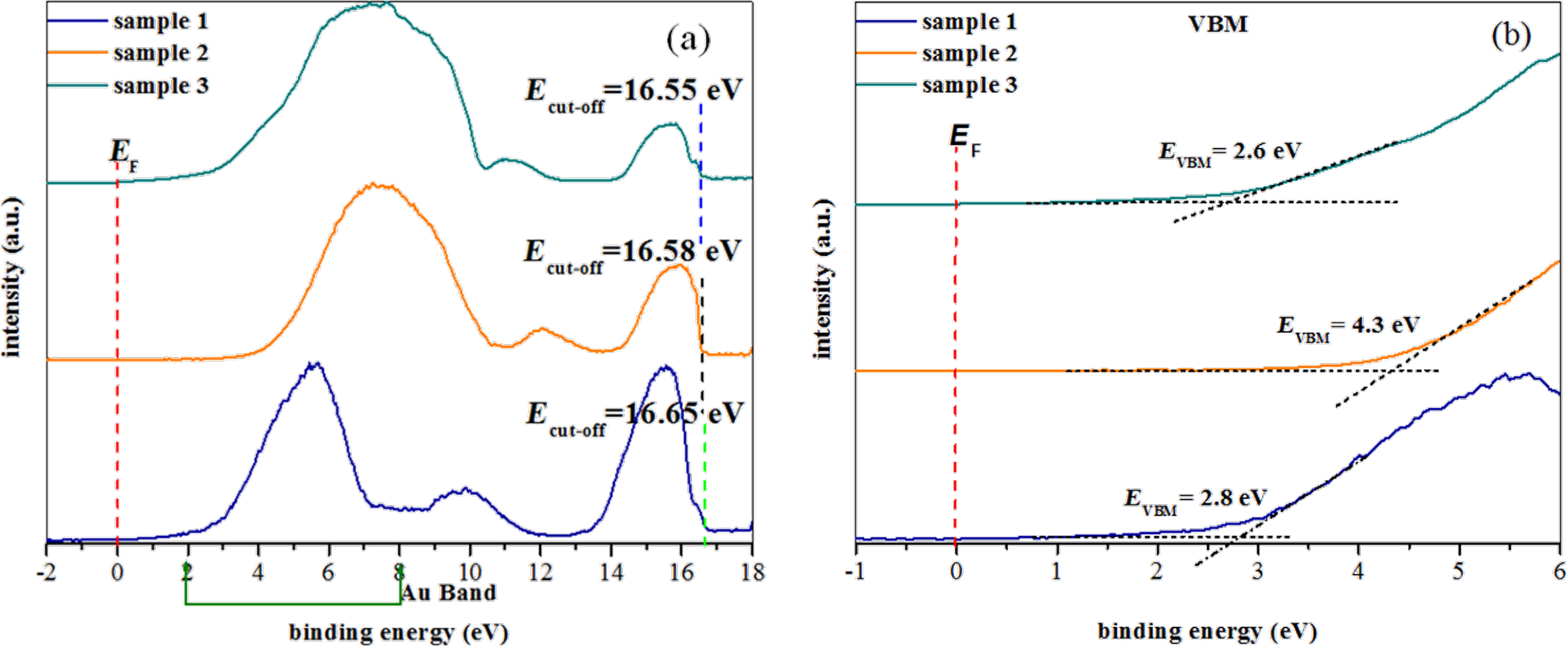
UPS spectra of (a) AuNPs deposited on APTES-functionalized glass substrates with 24 h APTES deposition and 8 h AuNPs deposition and (b) the valence band maximum of same samples obtained by linear extrapolations.

UPS spectra of AuNPs deposited on APTES-functionalized glass substrates are shown in Figure 6. All the samples for UPS analysis were taken from the same substrates that were studied by AFM (Figure 4, sample 1, 2 and 3). However, different UPS spectra are observed related to the orientation of APTES molecules on glass substrates (see Figure 2) that altered due to the attachment of AuNPs on the substrates. A broad band was observed at binding energies between 2 and 8 eV, which can be assigned to Au 5d band as the spin–orbit splitting of Au 5d_3/2_ and Au 5d_5/2_ are not observed [29]. The UPS spectra could be correlated with surface morphologies of the sample surfaces as shown in Figure 4. The UPS spectrum of sample 1 shows the characteristic Au 5d band feature, which is narrow and the flat band appearing at 7.2 to 8.8 eV due to incomplete attachment (observed from AFM image, Figure 4, sample 1) of AuNPs on APTES-functionalized glass substrate. The proper coverage of AuNPs (AFM image, Figure 4, sample 2) on the APTES-functionalized surface leads to the increasing band width of Au 5d feature with the spectrum intensity shifting from 5.6 eV (sample 1) to 7.3 eV (sample 2) with a subsequent narrowing of the flat band feature as observed in the UPS spectrum of sample 2. When AuNPs are completely adsorbed on the APTES-functionalized glass substrate (AFM image, Figure 4, sample 3), the flat band feature totally disappears and the Au 5d band width is also broader (see Figure 6a). The features observed at ca. 16 eV can be assigned as the background of the secondary electron emissions from the surfaces of AuNPs. The changes in the intensity of secondary electron emission due to the different surface morphologies of AuNPs on APTES-functionalized glass substrates. The work function also changed from 4.55 eV for sample 1 to 4.62 eV for sample 2 and 4.65 eV for sample 3, respectively. Due to different coverage of AuNPs on APTES-functionalized glass substrates, energy shift in VBM is observed (see Figure 6b).

### Effects of annealing of AuNPs deposited APTES-functionalized glass substrates in vacuum and air

**Figure 7:**
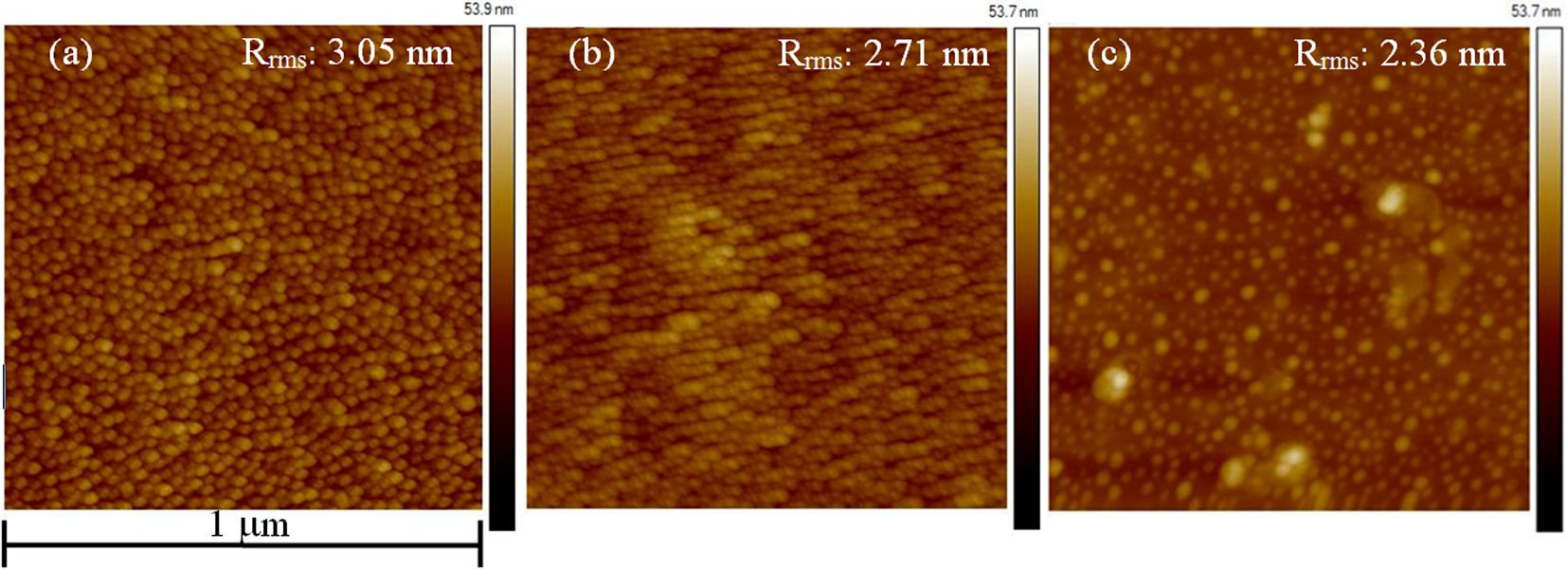
AFM images of Au NPs deposited on silanized glass substrates, (a) without annealing (as deposited) (b) annealed in vacuum (c) annealed in air at 600 °C for 1 h.

Annealing in vacuum and air (partial oxygen ambient) was also conducted on AuNPs deposited on APTES-functionalized glass substrates. AFM images of samples before and after annealing in vacuum and air at 600 °C for 1 h are shown in Figure 7. A slight change in surface morphology was observed after annealing in vacuum at 600 °C as some of the AuNPs realign themselves in a certain direction (see Figure 7b) due to the softening of the substrate at its glass transition point. Surface roughness, RMS (root mean square), was marginally reduced from 3.05 nm (before annealing) to 2.71 nm after annealing in vacuum (Figure 7a,b). However, upon annealing in air, AuNPs form discrete clusters scattered on the substrate surface, leading to a decrease in roughness of the surface with a calculated RMS value of 2.36 nm (Figure 7c). In this case, air was the driving force for AuNPs to migrate on soft glass substrate (the annealing temperature of 600 °C is slightly higher than glass transition temperature of about 557 °C) [30]. Annealing the glass substrate over its glass transition temperature permits it to come closer to thermodynamic equilibrium resulting in changes of its physical properties such as enthalpy, mechanical modulus, dielectric constant and specific volume [31]. The migration of AuNPs observed here was due to the changes in its physical properties. Upon annealing under vacuum, sample substrates reached to transition temperature, though due to the absence of driving force for migrating on the surface during annealing process leading to no specific reorganisation of AuNPs.

**Figure 8:**
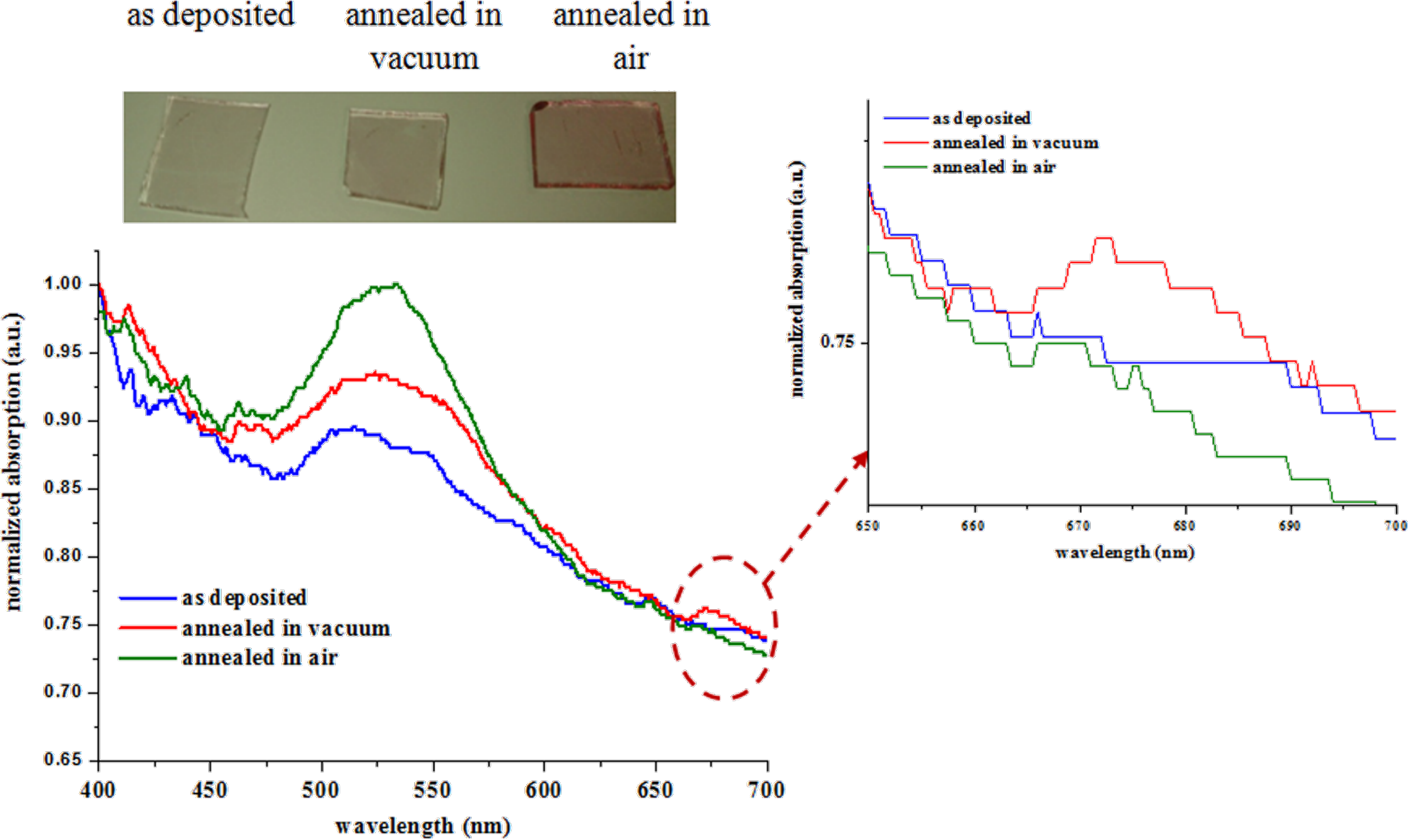
UV–visible absorption spectra of AuNPs deposited on APTES-functionalized glass substrate without annealing, annealed in vacuum and annealed in air; the inset shows the optical images of Au NPs deposited on APTES-functionalized glass substrate.

Figure 8 shows ultraviolet–visible absorption spectra of AuNPs deposited on APTES-functionalized glass substrates (before and after annealing in air and vacuum). Surface plasmon resonance (SPR) signals show band broadening in as deposited sample and the sample annealed in vacuum. A shoulder observed at 672 nm (marked as a region) for the sample annealed in vacuum is due to the alignment of the AuNPs on the glass substrate (see Figure 7b). Moreover, some AuNPs combined in a specific direction that led to rod-shaped structures where a longitudinal surface plasmon mode is observed. In contrast, annealing in air leads to samples with narrow SPR band and higher absorption intensity is obtained. The changes of the SPR band reveals color change of the corresponding samples varying from cyan to pink in appearance (Figure 8 inset). These changes of SPR band can be observed in surface profile (Figure 7). For instance, both as-deposited samples (without annealing) and samples annealed in vacuum show (Figure 7a,b) a dense distribution of AuNPs on the APTES-functionalized glass substrate with very small interparticle distance which is directly related to the coupling effect. The larger the interparticle distance, weaker is the coupling effect, that can lead to the narrowing of SPR band [32]. The as-deposited sample and sample annealed in vacuum provide stronger coupling effect, which results in broadening of SPR band. Sample annealed in air gives the largest interparticle distance amongst the samples studied in this work with weaker coupling effect and thus narrowing of SPR band can be observed as in Figure 8.

## Conclusion

The orientation of APTES molecules on glass surface plays an important role for interfacing of AuNPs. The deposition and orientation of APTES molecules on glass substrate surface were demonstrated by water contact angle measurements. UPS measurements further proved that different orientations of APTES molecules on glass substrate affect the self-organization of AuNPs on APTES-functionalized glass substrate. Surface topography of AuNPs deposited on glass surface was characterized by AFM which revealed the different coverage of AuNPs on self-assembled APTES glass substrates. Energy shift in VBM was also observed on APTES surface as well as AuNPs deposited on APTES surface. Furthermore, annealing in vacuum has demonstrated the aligning of AuNPs in a specific direction to form a rod-like structure which gives a longitudinal SPR mode in the UV–vis spectrum. However, annealing in air led to a larger inter-particle separation with a narrowing of the SPR band.

## Experimental

**Chemicals.** Hydrogen tetrachloroaurate (HAuCl_4_·3H_2_O) and trisodium citrate dihydrate (Na_3_C_6_H_5_O_7_·2H_2_O), 3-aminopropyltriethoxysilane were purchased from Sigma-Aldrich, USA. Acetone, absolute ethanol, sulfuric acid (H_2_SO_4_, 64%) and hyrogen peroxide (H_2_O_2_) were purchased from Merck, Germany. Deionized (DI) water (18.2 MΩ·cm) was used for preparation of solutions, synthesis and cleaning glass substrate. All the chemicals were used without further purification.

**Synthesis of gold nanoparticles (AuNPs).** 2 mL of 5 mM HAuCl_4_·3H_2_O in 50 mL of DI water was boiled under constant stirring. Upon boiling, 2.8 mL of 25 mM sodium citrate (Na_3_C_6_H_5_O_7_·2H_2_O) solution was then rapidly added upon constant heating, the color of the solution changed from pale yellow to ruby red. When no more change in color was observed, the container was quenched in an ice bath. The colloidal solution of as-synthesized AuNPs with the average diameter of ca. 10–15 nm in was ruby red and stable at room temperature.

**Self-organisation of AuNPs on APTES-functionalized glass substrates.** Glass substrates (1.3 cm × 1.3 cm) were sonicated in soap water, ethanol, acetone and deionized (DI) water for 20 min each and dried in an oven at 90 °C. Dried substrates were immersed in freshly prepared piranha solution (30% H_2_O_2_ and 96% H_2_SO_4_, 1:3) for 30 min at 70 °C. Subsequently, substrates were then thoroughly rinsed with DI water and dried in an oven at 90 °C. Then glass substrates were further cleaned with O_2_ plasma cleaner (Mini Flecto) for 5 min (80 W, 100% O_2_). Afterwards, the glass slides were immersed in 1% (v/v) solution of 3-aminopropyltriethoxysilane (APTES) in absolute ethanol for 24 h under room temperature. After that, APTES-functionalized glass substrates were sonicated with ethanol and DI water for 5 min each in order to remove any physisorbed APTES molecules. Prior to the deposition of AuNPs, all the glass substrates were dried in an oven at 90 °C for 1 h. As-prepared glass substrates were immersed in an aqueous solution of AuNPs for 8 h at room temperature to form monolayer of Au NPs on glass substrates. Then the glass substrates were rinsed with DI water and dried with N_2_ gas stream and stored in a desiccator until further use.

**High-temperature annealing of AuNPs deposited on glass substrates under vacuum and air**. For annealing in vacuum, samples were heated in ultra-high vacuum (10^−9^ mbar) environment at 600 °C for 1 h. For annealing in air, samples were annealed at 600 °C for 1 h in a commercial furnace.

**Characterization.** The surface morphology of AuNps self-organized on glass substrates were characterized by atomic force microscopy (AFM, Veeco di Multimode V). The operation was in contact mode using n-type antimony doped Si tips (TAP 525, Bruker) with a resonance frequency of 375–675 kHz at 0.5 Hz scan speed. For static contact angle measurements, the “sessile droplet” method was used to determine the surface wetting nature of APTES-functionalized glass surfaces using ThetaLite attention tensiometer (Biolin Scientific, Sweden). 5 µL water droplets were used and water contact angle was measured at five random locations on each sample surface. X-ray photoelectron spectroscopy (XPS) measurements were carried out using Omicron Nanotechnology system with Al Kα radiation (1486.6 eV). All XPS measurements were conducted in ultrahigh high vacuum conditions of 2 × 10^−10^ mbar. In order to reduce surface charging effects, all the measured samples were flooded with electrons for charge compensation during the XPS measurements. The binding energies were calibrated with respect to adventitious C 1s feature at 284.6 eV. XPS spectra were de-convoluted to individual components using Gaussian–Lorentzian function after background subtraction with Shirley function in Casa XPS software. The band structures of the samples (glass substrates, APTES-functionalized glass substrates and AuNPs deposited on APTES-functionalized glass substrates) were studied by UPS using He-I lamp with energy of 21.2 eV. UV–vis spectrophotometer (Perkin Elmer, Lambda 25) was used to study the optical properties of AuNps deposited on APTES-functionalized glass substrates in the wavelength range of 400–700 nm.
